# Efficient cellular and humoral immune response and production of virus-neutralizing antibodies by the Hepatitis B Virus S/preS1^16-42^ antigen

**DOI:** 10.3389/fimmu.2022.941243

**Published:** 2022-07-22

**Authors:** Ana-Maria Pantazica, Mihaela-Olivia Dobrica, Catalin Lazar, Cristina Scurtu, Catalin Tucureanu, Iuliana Caras, Irina Ionescu, Adriana Costache, Adrian Onu, Jihong Liu Clarke, Crina Stavaru, Norica Branza-Nichita

**Affiliations:** ^1^ Department of Viral Glycoproteins, Institute of Biochemistry of the Romanian Academy, Bucharest, Romania; ^2^ Immunology Laboratory, “Cantacuzino” Medico-Military National Research Institute, Bucharest, Romania; ^3^ Division of Biotechnology and Plant Health, NIBIO - Norwegian Institute for Bioeconomy Research, Ås, Norway

**Keywords:** HBV, vaccine design, SVP, antigens, chimeric proteins, adjuvants

## Abstract

Despite the availability of improved antiviral therapies, infection with Hepatitis B virus (HBV) remains a3 significant health issue, as a curable treatment is yet to be discovered. Current HBV vaccines relaying on the efficient expression of the small (S) envelope protein in yeast and the implementation of mass vaccination programs have clearly contributed to containment of the disease. However, the lack of an efficient immune response in up to 10% of vaccinated adults, the controversies regarding the seroprotection persistence in vaccine responders and the emergence of vaccine escape virus mutations urge for the development of better HBV immunogens. Due to the critical role played by the preS1 domain of the large (L) envelope protein in HBV infection and its ability to trigger virus neutralizing antibodies, including this protein in novel vaccine formulations has been considered a promising strategy to overcome the limitations of S only-based vaccines. In this work we aimed to combine relevant L and S epitopes in chimeric antigens, by inserting preS1 sequences within the external antigenic loop of S, followed by production in mammalian cells and detailed analysis of their antigenic and immunogenic properties. Of the newly designed antigens, the S/preS1^16–42^ protein assembled in subviral particles (SVP) showed the highest expression and secretion levels, therefore, it was selected for further studies *in vivo*. Analysis of the immune response induced in mice vaccinated with S/preS1^16–42^- and S-SVPs, respectively, demonstrated enhanced immunogenicity of the former and its ability to activate both humoral and cellular immune responses. This combined activation resulted in production of neutralizing antibodies against both wild-type and vaccine-escape HBV variants. Our results validate the design of chimeric HBV antigens and promote the novel S/preS1 protein as a potential vaccine candidate for administration in poor-responders to current HBV vaccines.

## Introduction

Chronic Hepatitis B Virus (HBV) carriers account for approximately 3.5% of the world population, making this pathogen one of the leading global health problems (1, 2). Up to 40% of these individuals will develop liver cirrhosis and hepatocellular carcinoma, resulting in more than 800,000 deaths yearly (3). Although current HBV therapies reduce the viral load and the risk of disease progression in a significant number of treated people, they do not affect the nuclear reservoir of covalently closed circular (ccc) DNA replication from within infected hepatocytes. Therefore, patients must receive lifelong treatment to prevent viral rebound, which often results in adverse effects and increases the probability for the development of viral resistance.

To achieve the ambitious goal of eliminating HBV infection as a public health threat by 2030, set by WHO (4), combined efforts must address increasing diagnostic and treatment coverage, as well as more efficient prophylactic interventions, including vaccination (5). Current HBV subunit vaccines produced in yeast have significantly contributed to reduce HBV incidence worldwide, since they were first marketed in 1986 (6). These vaccines are based on the ability of the HBV small (S) envelope protein to self-assemble into highly immunogenic, 20 nm-diameter subviral particles (SVPs), even when expressed on its own. The 3-dose administration schedule of the recombinant S vaccines ensures sufficient levels of protective antibodies in more than 90% of healthy adult recipients, depending on their age (7). While the efficacy of this immune response has undoubtedly been demonstrated, its persistence is still a matter of debate, with a number of studies indicating that certain individuals may require a vaccine booster dose to maintain seroprotective antibody levels (8). In addition, complete failure to respond to HBV vaccination remains a major health problem, especially in highly endemic areas, which must be urgently tackled by developing more immunogenic vaccines (9). In this respect, targeting the innate immune response by using the Toll-like receptor-9 activator (TLR-9), cytosine phosphoguanosine (CpG) 1018, has significantly improved the immunologic properties of the HBV vaccine and a two-dose formulation has recently been approved for use in adults (10, 11).

Another strategy has considered inclusion of all three HBV envelope proteins, S, medium (M) and large (L), produced in mammalian cells, in a third-generation vaccine formulation (12). Recent clinical trials concluded in 2021, revealed that this novel HBV vaccine is highly immunogenic in adults, including the non-responders to conventional S only-based vaccines, which prompted FDA approval for its use in adults (13, 14).

Other promising vaccine designs take advantage of the SVP scaffold to create chimeric antigens which contain relevant structural and functional epitopes of the L and M proteins (15–20). Using this strategy, we have previously created a novel chimeric HBV antigen, by inserting the 21–47 amino acid (aa) sequence of the preS1 domain of the L protein (genotype D) between aa 126/127 within the antigenic loop (AGL) of the S protein. The S/preS1^21–47^ chimeric antigen triggered superior humoral and cellular immune response in immunized mice when compared with the wild-type (WT) S protein and a mixture of anti-S and anti-preS1 antibodies with HBV neutralizing capacity. However, these very promising results were overshadowed by the limited production of S/preS1^21–47^–SVPs in different systems, including mammalian cells and plants (15, 20). Given the crucial role of the N-terminal 47 aa of the preS1 domain in binding the sodium taurocholate cotransporting polypeptide (NTCP), the HBV receptor (21), its reactivity to virus neutralizing antibodies (22–24) and the superior immunogenic properties of the S/preS1 chimeric antigens (15), we believe this strategy deserves deeper scrutiny.

In this study, we further optimized the design of chimeric S/preS1 antigens, by varying the length of the preS1 epitope or that of the recipient S protein, in order to improve SVP assembly and secretion and potentially elicit high levels of HBV neutralizing antibodies. Of the newly designed constructs, the S/preS1^16–42^ antigen retained the ability to assemble into SVPs and exhibited significantly improved expression and secretion levels when compared with the chimeric antigen generated previously. Analysis of the immune response induced in mice following vaccination with S/preS1^16–42^- and S-SVPs, respectively, demonstrated the higher immunogenicity of the former and its ability to produce neutralizing antibodies against both WT and vaccine-escape HBV variants. Our results suggest that the newly developed HBV antigen could be a strong candidate for administration in non-responders to the standard, S-based vaccines.

## Materials and methods

### Plasmids

The previously described pCi-HBV-S/preS1^21-47^ plasmid encoding the chimeric S/preS1^21-47^ antigen (15) was used as a template to generate novel constructs encoding preS1 epitopes comprising aa 10-36, 16-42 and 16-47, respectively, inserted within the AGL domain of S protein, between aa 126 and 127 (genotype D). The 16-47 preS1 sequence is slightly longer than the maximum length of insertions (27 aa) known to be tolerated by the AGL loop without disturbing its folding and stability (25). To potentially compensate for this length, an additional construct was generated as control, which contains the same epitope inserted between aa 126 and 132 of the AGL domain (i.e. the aa sequence 127-131 was deleted).

The new plasmids, pCi-HBV-S/preS1^10-36^, pCi-HBV-S/preS1^16-42^, pCi-HBV-S/preS1^16-47^ and pCi-HBV-SΔ^127-131^/preS1^16-47^ were generated by using the Q5 Site-Directed Mutagenesis Kit (NEB) and specific primers (**Table 1**). The same mutagenesis approach with the pGEM-4Z-HBV 1.3 WT (gift from Wang-Shick Ryu, Addgene, #65459) plasmid as a template were exploited to produce virus particles containing the representative G145R vaccine-escape mutation within the S domain. The primers used are described in **Table 1**.

**Table 1:** Templates and primers used to generate the novel S/preS1 antigens and the HBV vaccine-escape mutant.

Antigens	Template	Forward primer	Reverse primer
S/preS1^16-47^	S/preS1^21-47^	5′GACCACCAGTTGGATCCAGCCTTCAGAGCAAACACCGC3′	5′AGTCATGCAGGTCCGGCATGGTCCCG3′
S/preS1^16-42^	S/preS1^16-47^	5′ACTGCTCAAGGAACCTCTATGTATCCCTC3′	5′TGGCCAGGTGTCCTTGTTGGG3′
SΔ^127-131^/preS1^16-47^	S/preS1^16-47^	5′TCTATGTATCCCTCCTGTTGCTGTACCAAACCTTCG3′	5′TACCTTGTTGGCGTCTGGCCAGG3′
S/preS1^10-36^	1. S/preS1^16-42^	5′TTCTTTCCCGACCACCAGTTGGATCCAG3′	5′TCCCAGAGGAGTCATGCAGGTCCGG3′
	2. S/preS1^10-42^	5′ACTGCTCAAGGAACCTCTATGTATCCC3′	5′GGGATTGAAGTCCCAATCTGGATTTGC3′
HBV-S^G145R^	HBV 1.3-mer WT (Addgene #65459)	5′ACCTTCGGACAGAAATTGCAC 3′	5′TTGGTACAGCAACAGGAGG 3′

### Cell lines and virus production

HEK293T cells were cultured in Dulbecco’s Modified Eagle Medium (DMEM) (Gibco) supplemented with 10% fetal bovine serum (FBS, Gibco) and 1% non-essential amino acids (NEAA) (Gibco). HepG2^hNTCP^ cells used for neutralization assays (gift from Professor Stephan Urban, German Center for Infection Research, University of Heidelberg) were cultured in DMEM supplemented with 10% FBS, 1% NEAA and 2.5 µg/mL puromycin (Invitrogen). HepG2.2.2.15 cells (gift from Dr. David Durantel, INSERM U871, Lyon, France), used for production of WT-HBV particles were cultured as previously described (26). All cell lines were maintained at 37°C in an incubator with 5% CO_2_. HBV secreted from HepG2.2.2.15 cells was concentrated *via* ultracentrifugation on a 20% sucrose bed and then quantified by qPCR, as previously described (27). Stocks of HBV particles were also obtained following transfection of Huh7 cells with pGEM-4Z-HBV 1.3 WT or pGEM-4Z-HBV 1.3 G145R, by using Lipofectamine 3000 (Invitrogen), according to the manufacturer’s instructions. Cell media were collected between days 7 and 12 post-transfection, concentrated by ultracentrifugation on a 20% sucrose bed and quantified by qPCR.

### Production of HBV antigens in HEK293T cells

HEK293T cells were seeded in 6-well plates and then transfected with pCi plasmids encoding the chimeric S/preS1 antigens described above or the S protein, by using Lipofectamine 3000 (Invitrogen) according to the manufacturer’s instructions. Cells transfected with the empty pCi vector were used as a control. The extracellular media and cells were collected at 72 h post-transfection. For large scale production, HEK293T cells were seeded in 1700 cm^2^ ribbed-surface roller bottles and cultured in a Roll-In CO_2_ Control incubator (Wheaton) as described previously (28). The extracellular media was then collected at 4- and 10-days post-transfection and used for further purification.

### Purification of HBV antigens

The supernatants of HEK293T cells expressing the novel HBV antigens or the S protein were 20-fold concentrated *via* ultracentrifugation on a 20% sucrose-bed, for 5 h at 30,000 rpm (SW 32 Ti, Beckman Coulter). Concentrated samples were layered on a step sucrose gradient (15-60%) and subjected to ultracentrifugation at 32,000 rpm (SW 42Ti, Beckman Coulter) for 16 h. Fractions were collected and analyzed for the presence of antigens *via* ELISA, by using the Monolisa HBsAg Ultra kit (BioRad), that detects secreted S, M and L proteins assembled in SVPs, collectively known as the HBsAg. HBsAg-positive fractions were pooled, dialyzed against PBS and subjected to size-exclusion chromatography on the CaptoCore 400 resin (Cytiva), by gravitational flow. The presence of the viral antigens in flow-through fractions was confirmed by western blot or ELISA (for the S protein). Positive fractions were then pooled and concentrated on Amicon-100 kDa (Millipore) columns. HBV antigens were quantified by ELISA and western blot as previously described (15). The BCA assay (Thermo Scientific-Pierce) was used to determine the total protein content in each sample fraction, to estimate purification yields.

### Western blot

Protein samples were separated by sodium dodecyl-sulfate polyacrylamide gel electrophoresis (SDS-PAGE) and then transferred onto nitrocellulose membranes. The membranes were blocked in 10% skimmed dry milk in PBS for 1 h, followed by successive incubations with mouse anti-preS1 antibodies (sc-57762, Santa Cruz Biotechnology, 1:1000) overnight at 4°C and HRP-conjugated mouse-IgGκ binding protein (sc-516102, Santa Cruz Biotechnology, 1:10,000) for 1 h at room temperature (RT). Proteins were visualized by using an enhanced chemiluminescence assay (ECL, Thermo Scientific-Pierce). Protein band intensity was determined by using the Image J software (National Institutes of Health) and HBV-S/preS1 protein contents were quantified by western blot using a standard curve of commercial L protein, as described before (preS1 antigen, Beacle) (15).

### Pulse-chase and immunoprecipitation

HEK293T cells were seeded in 6-well plates and transfected with pCi-HBV-S/preS1 constructs described above or an empty pCi vector, by using Lipofectamine 3000 (Invitrogen) according to the manufacturer’s instructions. At 24 h post-transfection, the medium was removed and the cells were washed three times with PBS and incubated with RPMI-1640 modified medium (Sigma Aldrich), without methionine, cysteine, and L-glutamine, supplemented with GlutaMAX (Sigma-Aldrich), for 1 h at 37°C. Cells were pulse-labeled with 18 µCi/well of ^35^S-labeled methionine/cysteine (EXPRESS35S Protein Labeling Mix, [^35^S] EasyTag, Perkinelmer) for 30 min at 37°C and chased in RPMI-1640 supplemented with 10 mM methionine. Cells and media were collected at 0 and 3 h post-labeling. Radiolabeled cells were lysed in lysis buffer (10 mM Tris-HCl, pH 7.5, 150 mM NaCl, 2 mM EDTA, and 0.5% Triton X-100) for 30 min, on ice, then subjected to centrifugation at 14,000 × *g* for 10 min, at 4°C. Clarified cell lysates and media were incubated with a mixture of horse anti-S (ab9193, Abcam, 1:100) and mouse anti-preS1 (sc-57762, Santa Cruz Biotechnology, 1:100) antibodies, overnight, at 4°C. Afterwards, the samples were incubated with 15 μL of Protein G-Sepharose beads (Sigma Alrdrich) for 3 h, at 4°C, under gentle agitation. The bound proteins were washed seven times with washing buffer (10 mM Tris-HCl, pH 7.5, 150 mM NaCl, 2 mM EDTA, and 0.1% Triton X-100), pelleted by centrifugation at 1,500 × *g*, for 2 min and then eluted by boiling in Laemmli buffer containing DTT, for 10 min, at 95°C. The samples were then separated by SDS-PAGE and visualized by autoradiography.

### Animals and immunization

Animal experiments were conducted in accordance with standards set forth in the Council Directive 86/609/EEC and national legislation, approved by the national designated authority, ANSVSA, number 488/22.01.2020. In brief, 4 groups of 5, 6-8-week-old female Balb/c mice were immunized three times intramuscularly, at a 14-day interval. The antigen groups received 5 μg of either HVB-S/preS1^16-42^ or HVB-S in combination with 0.25 mg/mL Al(OH)_3_ (Alhydrogel, *In vivo*Gen), as adjuvant, per injected dose. The control groups received 5 μg of background HEK293T proteins (i.e. proteins from mock-transfected cell supernatants subjected to identical purification steps as the HBV antigens) in the presence of 0,25 mg/mL Al(OH)_3_, or the adjuvant alone. The total volume of the administered dose was 50 µL/mouse. Blood samples were collected by retro-orbital bleeding prior to priming and at days 27 and 49 post-immunization. Following clotting, samples were centrifuged at 14, 000 × *g* for 10 min at RT and the resulting sera were stored at -80°C until further analysis. The mice were sacrificed on day 56 under anaesthesia, with final bleeding. Mice sera and spleens were collected for the functional tests.

### Analysis of the humoral immune response by ELISA

Flat-bottom 384-well MediSorp plates (Sigma-Aldrich) were coated with 20 μL/well of UV-inactivated HBV suspended in PBS (containing 0.4 μg/mL of HBsAg), overnight at 4°C, followed by extensive washing in PBS containing 0.05% Tween 20 (Sigma-Aldrich). The plates were further treated with 5% nonfat dry milk in PBS (Bio-Rad), for 1 h, at RT, then washed again, as above. A 40 μL volume of two-fold serially diluted serum samples (1:200-1:6400) in PBS containing 5% nonfat dry milk in PBS was added to the antigen-coated plates and incubated for 2 h, at RT. The plates were washed again before incubation with HRP-conjugated goat anti-mouse IgG (1:6000) or IgG1/IgG2a (1:1000) (Southern Biotech), diluted in PBS containing 5% nonfat dry milk, for 2 h, at RT. The enzymatic reaction was initiated by incubation with the 3,3′,5,5′-Tetramethylbenzidine (TMB) substrate (R&D Systems) according to the manufacturer’s instructions and optical densities (ODs) were read at 450 nm in a Thermo Scientific Multiskan FC microplate reader. Normalization across ELISA plates were performed by using serial dilutions of the pool of all the immune sera from day 56 as an internal standard on each plate. A 4-parameter logistic regression model was fitted to the measured values (29). Values of sample dilutions with an estimated error below 30% were averaged by arithmetic mean. Values of samples falling below the detection range were assigned a value of zero. A calculated value of 1 indicates antibody reactivity equal to that of the pooled sera. All calculations were performed in R version 3.1.1(x) using the calibFit 2.1.0 package (30). Statistical analysis was carried out to reveal the differences between groups by using the Wilcoxon rank-sum test.

### Analysis of the cellular immune response by cytokine-multiplex immunoassay

Splenocytes isolated from immunized mice were seeded at 1×10^6^ and 0,25×10^6^ cells/well, in triplicate, in RPMI-1640 media containing 10% fetal calf serum (FCS) (Gibco) and 2-mercaptoethanol (Sigma-Aldrich). Cells were then stimulated with either UV-inactivated HBV (containing 5 µg/mL HBsAg) or the known cellular immune response activators CD3/CD28 (0.5 μg/mL, R&D Systems) and ConA (5 μg/mL, Sigma-Aldrich), as positive controls, for 36 h at 37°C, in a 5% CO_2_ incubator. Unstimulated, PBS-treated cells were also included in the experiment as a negative control. Cell culture supernatants were tested for relevant cytokine levels (IL-4, IFN-γ, IL-2, TNF-α, IP10, IL-10, MCP1, MIP1a, IL-13, IL-5, IL-1b, IL-6, IL-17, IL-23), by using the Mouse Premixed Multi-Analyte kit LXSAMS-14 (R&D Systems), according to the manufacturer’s instructions. Briefly, 50 μL of either standard or mice-derived samples were added to each well together with the diluted magnetic microparticle cocktail and incubated for 2 h, at RT, with gentle shaking. Plates were washed three times in washing buffer and then incubated with 50 μL/well of diluted biotin-antibody cocktail, for 1 h, at RT, with gentle shaking. Following washing, the plates were incubated with 50 µL/well of diluted Streptavidin-PE for 30 minutes at RT and washed again. The plate reading was performed on the Luminex 100ä platform (Athena Multi-Lyte Instrument) and data were processed with the Luminex 100 IS 2.3 Software. The concentration for each cytokine in pg/mL is shown. Clusters and dendrograms were automatically generated in R studio version 1.4.1103 by using the latticeExtra and dendextend packages (hclust by manhattan method and dendrograms by wald.D2 method).

### Functional characterization of anti-HBV antibodies

HEK293T cells were transfected with pCi-HBV-S, pCi-HBV-L, or a previously generated plasmid, pCi-HBV-S^Δ127-150^/preS1^21-47^ in which the AGL domain of the S protein was replaced by the preS1 aa 21-47, resulting in lack of recognition by the anti-S antibodies (15). Cell lysates were used to coat 96-well plates (30 µg of total protein/well), overnight at 4°C. Plates coated with mock-transfected HEK293T cell lysates were used as control. Plates were washed five times in PBS supplemented with 0.1% Tween-20 (Sigma Alrdrich) and blocked in 10% skimmed dried milk in PBS, for 1 h, at RT. Plates were washed again and incubated with serial dilutions (1:200, 1:400, 1:800, 1:1600, 1:3200) of sera from mice immunized with HBV-S/preS1^16-42^, for 3 h, at RT. Following extensive washing in PBS supplemented with 0.1% Tween-20, samples were incubated with HRP-conjugated mouse-IgGκ binding protein (sc-516102, Santa Cruz Biotechnology, 1:10,000), for 1 h, at RT. Detection was performed by incubation with TMB for 30 min. The OD was read at 450 nm in a Mithras Microplate Reader, following addition of 2 N H_2_SO_4_ to stop the enzymatic reaction.

### Neutralization of HBV infection by the immune sera

Pre-immune sera and sera from mice immunized with the HBV-S, HBV-S/preS1^16-42^, as well as background proteins, collected at day 56 post-immunization, were diluted 1:50. Due to the limited volume available and the large number of experiments requiring this control, the pre-immune sera were pooled per mice group. The viral inoculum corresponding to 100 genome equivalents (GEq)/cell of either WT HBV or the vaccine escape HBV-S^G145R^ was pre-incubated with diluted sera, in complete DMEM supplemented with 4% polyethylene glycol (PEG, Sigma Aldrich), for 1 h, at 37°C. HepG2^hNTCP^ cells seeded in 48-well plates were infected with the sera-pretreated HBV inoculum, for 16 h, at 37°C. To control for specific inhibition of HBV infection, Myrcludex B (1 µM, Pepscan) was added to cells 3 h prior to the viral inoculum. Cells incubated with HBV only were used as control for the maximum level of infection. Cells were then washed three times with PBS and incubated with complete media supplemented with 2.5% DMSO (AppliChem). The medium was changed every two days until day 7 post-infection. The media accumulated from days 7 to 11 post-infection were collected and tested for the HBeAg levels, by using the Monolisa HBe Ag-Ab PLUS kit (BioRad). Data are shown as percentage of infection in the presence of post-immune sera from infectivity in the presence of the pre-immune sera, at the same dilution. Statistical analysis was performed by using the Mann–Whitney U test using the GraphPad Prims version 6 software.

## Results

### Antigen design

**Figure 1:**
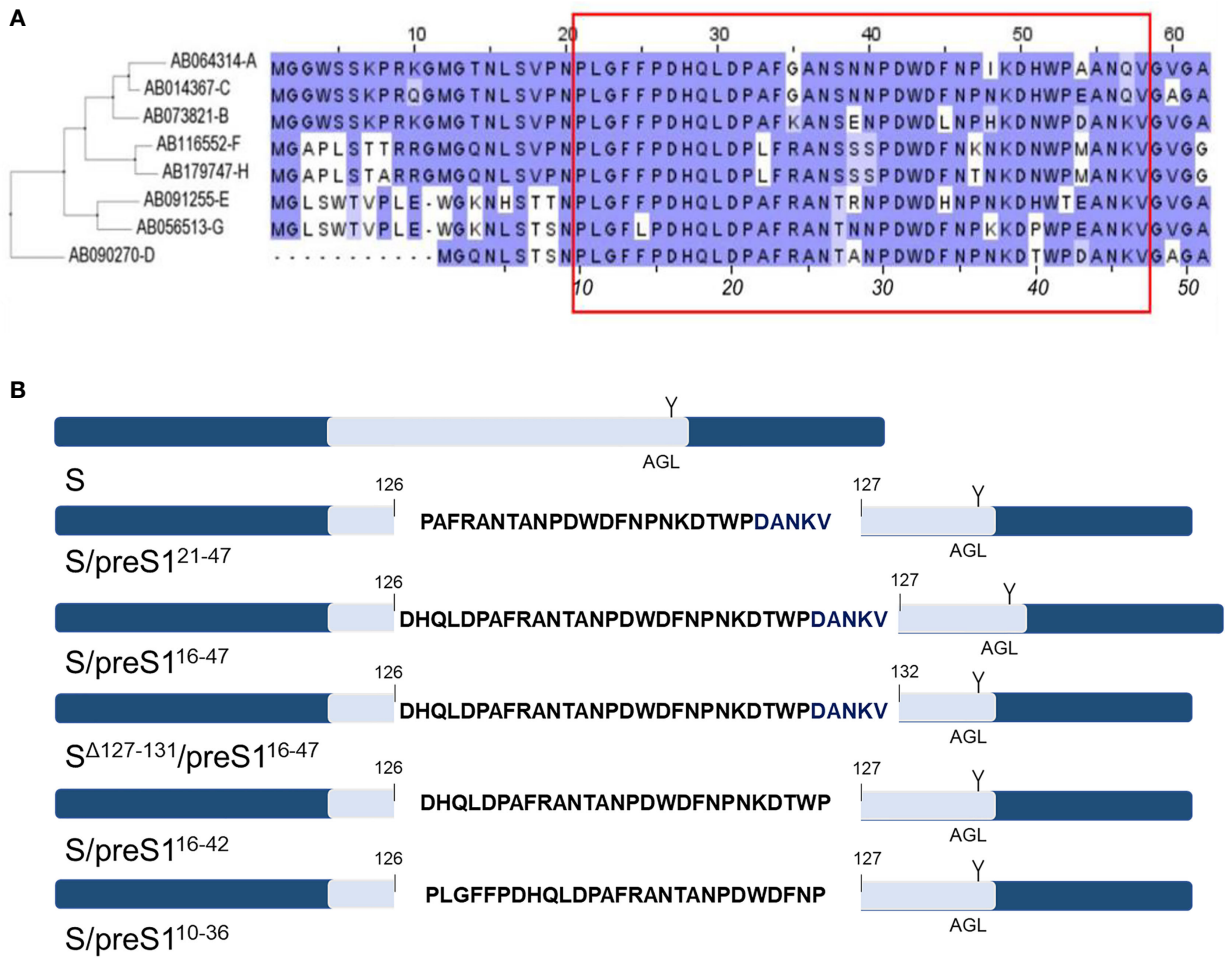
Design of novel HBV S/preS1 antigens. **(A)** The amino acid sequences corresponding to the preS1 region of prevalent HBV isolates were aligned using the Blosum62 matrix. Genotypes A to H are indicated on the left side according to their GenBank annotations. The red box illustrates the preS1 antigenic region which can generate virus neutralizing antibodies (numbers in italics apply to genotype D). **(B)** preS1-derived sequences were inserted between amino acids 126/127 or 126/132 of the antigenic loop (AGL, depicted in light blue) of the S protein. The unique N-glycosylation site at position N146 of the S protein is also shown (Y).

The ability of the current S-based vaccines to trigger an efficient virus-neutralizing immune response relies on the presence of strongly immunogenic B-cell epitopes, located between aa 122-150 of the S protein, collectively known as the “a” determinant of the AGL (31), that is shared by all HBV genotypes. Notably, the NTCP-binding site of the preS1 domain of the HBV-L protein is also a target of virus neutralizing antibodies (23, 32) and a map of the epitopes with reactivity to antibodies with potent HBV-neutralizing activity has recently been revealed (24). Consistent with these findings, our previously reported chimeric S/preS1^21-47^ antigen, containing the aa 21-47 of preS1 (genotype D) inserted between aa 126 and 127 of the S protein, elicited stronger humoral immune response in vaccinated mice when compared with the S protein and antibodies against both the carrier and the inserted peptide, with inhibitory activity of HBV infection *in vitro* (15, 20). Although this chimera retained the ability of the WT S protein to assemble into SVPs, the production capacity in mammalian or plant cells remained modest, hampering large scale applications. Therefore, our novel design to optimize the S/preS1 chimeric antigens has considered the S/preS1^21-47^ protein as a reference. Our strategies aimed to (i) improve protein stability and thus yields, by reducing the hydrophobicity of the inserted preS1 sequence and (ii) obtain antibodies with potential broad-neutralizing activity, by inserting a preS1 region containing more conserved epitopes across genotypes. Following alignment of the preS1 sequences of prevalent HBV isolates (**Figure 1A**), preS1 sequences comprising aa 10-36, 16-42 and 16-47 (genotype D) were selected for insertion within the AGL domain of the S protein, between aa 126 and 127. These regions partially overlap the original 21-47 sequence, extending it at the N-terminus by addition of conserved aa (**Figure 1B**). Deletion of aa 127-131 at the insertion site within the S protein was also performed in a control construct, to compensate for the introduction of the longer, 16-47 preS1 sequence. This experiment aimed to determine whether maintaining an appropriate length of the AGL domain at the expense of its integrity influences the chimeric protein stability and antigenicity (25). Analysis of the hydrophobicity profile of the selected preS1 inserts revealed that sequences spanning aa 16-42 and aa 16-47 are less hydrophobic than the aa 21-47 reference (**Table 2**).

**Table 2:** The hydrophobicity of the inserted preS1 sequences was calculated using the Peptide Hydrophobicity/Hydrophilicity Analysis Tool (https://www.peptide2.com/N_peptide_hydrophobicity_hydrophilicity.php).

HBV-S/preS1 chimera	Hidrophobicity of inserted preS1 sequences
S/preS1^21-47^	48.15%
S/preS1^16-47^	43.75%
SΔ^127-131^/preS1^16-47^	43.75%
S/preS1^16-42^	44.44%
S/preS1^10-36^	55.56%

### Expression and characterization of the novel S/preS1 antigens in HEK293T cells

**Figure 2:**
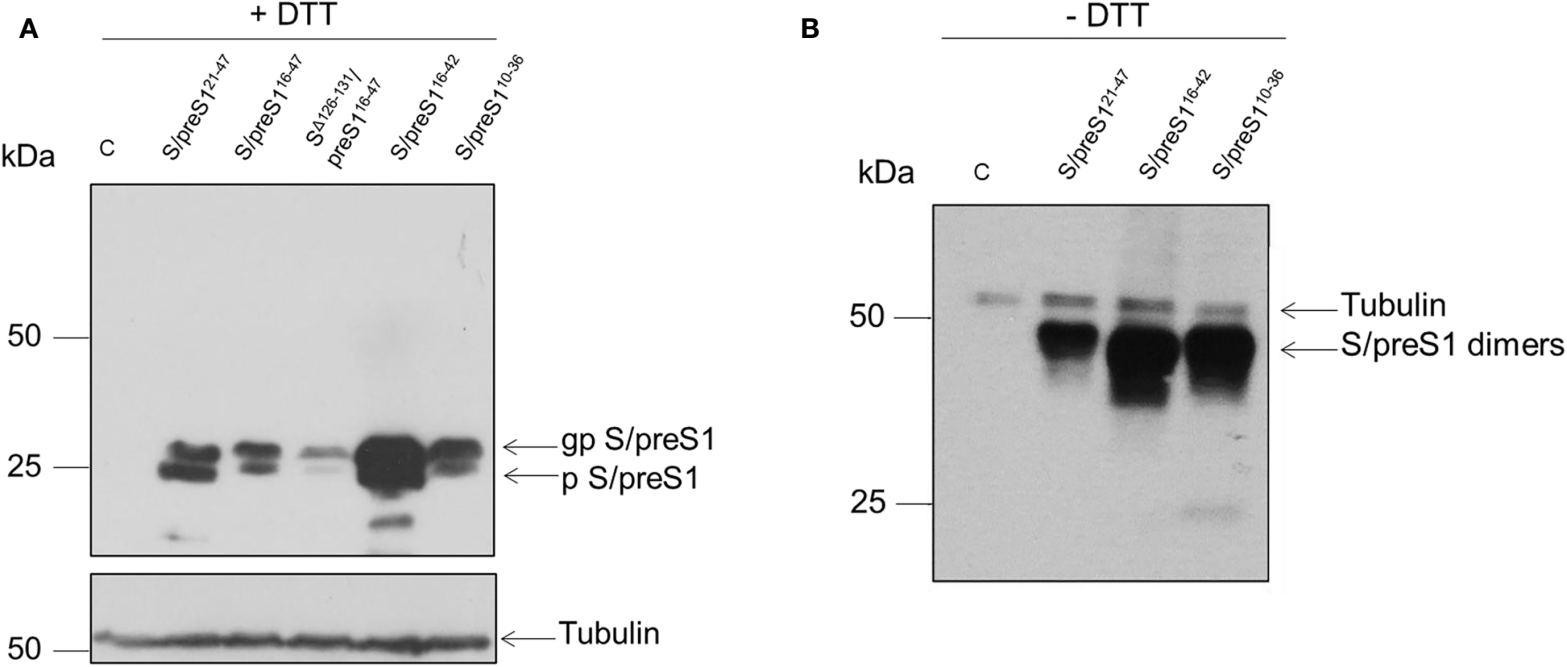
Expression of novel HBV S/preS1 antigens in mammalian cells. HEK293T cells were transfected with pCi plasmids encoding the indicated antigens or the empty pCi plasmid as control (C). **(A)** Cell lysates were analyzed by western blot under reducing (+DTT) conditions. **(B)** Cell lysates were analyzed by western blot under non-reducing (-DTT) conditions. Glycosylated (gp) or non-glycosylated (p) S/preS1 proteins were detected with anti-preS1 antibodies. Tubulin was used as a loading control. Representative images are shown (n=3).

To determine the expression levels of the newly designed antigen constructs, HEK293T cells were transfected with the corresponding plasmids, including that encoding for the previously developed S/preS1^21-47^ antigen, for comparison. At 72-h post-transfection, the cells were lysed, analyzed for the total protein content and samples containing equal amounts of protein were subjected to SDS-PAGE under reducing (+DTT) conditions. The chimeric S/preS1 antigens were detected by western blot, using anti-preS1 antibodies. The DTT treatment revealed the presence of S/preS1 monomers resolved in two bands with an apparent molecular weight of ~24 and ~29 kDa, corresponding to the non-glycosylated (p) and glycosylated (gp) polypeptides respectively, which suggests appropriate N-glycosylation of the S domain (**Figure 2A**). Interestingly, of the tested constructs including the former S/preS1^21-47^ antigen, the S/preS1^16-42^ antigen had the highest expression level (**Figure 2A**). Insertion of the longer (32 aa) preS1 sequence resulted in poor expression of the corresponding S/preS1^16-47^ and SΔ^127-131^/preS1^16-47^ proteins confirming the limitation of the S protein to accommodate large epitopes at the 126/127 insertion site (25). In addition, insertion of the same epitope in a shorter AGL, proved deleterious for the expression of the SΔ^127-131^/preS1^16-47^ antigen, likely affecting the folding and hence stability of the protein (**Figure 2A**).

The ability of the best expressed antigens to acquire intermolecular disulfide bonds, the first step in SVP assembly, was next analyzed by SDS-PAGE under non-reducing (-DTT) conditions, followed by western blot, as above. As shown in **Figure 2B**, all tested chimeric constructs were able to form dimers with an apparent molecular weight of ~50 kDa. The results confirmed the highest expression of the S/preS1^16-42^ antigen observed previously.

**Figure 3:**
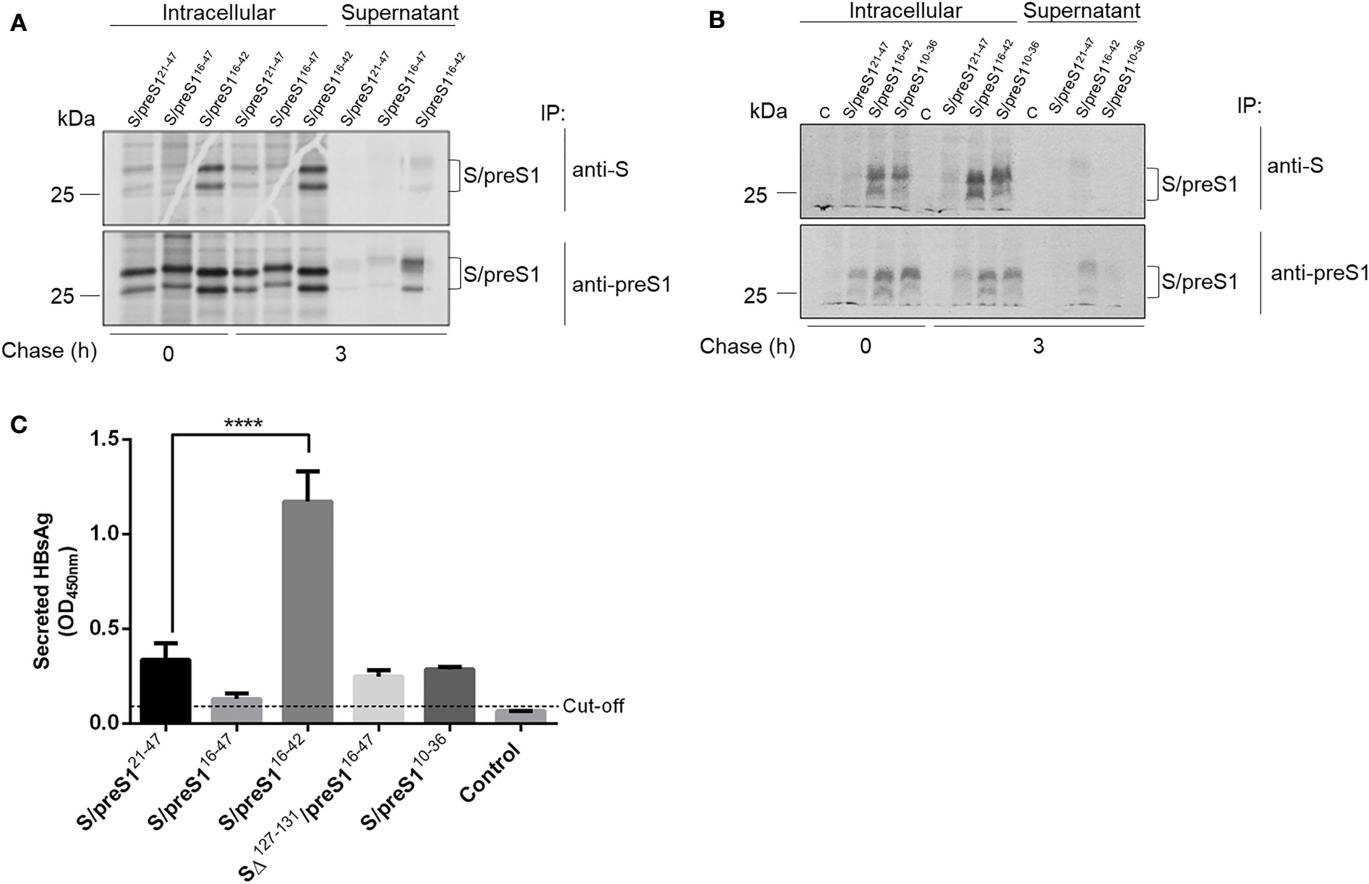
Antigenicity of novel HBV S/preS1 antigens produced in mammalian cells. **(A, B)** HEK293T cells transfected with pCi plasmids encoding the indicated antigens or the empty pCi plasmid as control (C) were pulse-labelled with ^35^S-protein labelling mix for 30 min and chased for 3 h. Cell lysates and supernatants were immunoprecipitated with either anti-S, or anti-preS1 antibodies and bound proteins were separated by SDS-PAGE and visualized by autoradiography. **(C)** HEK293T cells were transfected with pCi plasmids encoding the indicated antigens or the empty pCi plasmid as control (Control) for 72 h. Secretion of the HBsAg was quantified by ELISA in cell supernatants and represented as optical density values measured at 450 nm (n=3). Data are represented as means ± SD; Student’s unpaired t test (**** p < 0,0001).

We further investigated the antigenicity and secretion of the S/preS1 antigens by pulse-chase and immunoprecipitation with anti-S and anti-preS1 antibodies. Crude extracts and supernatants of ^35^S-labeled cells expressing the chimeric proteins were immunoprecipitated with conformation-dependent (anti-S) or independent (anti-preS1) antibodies followed by SDS-PAGE and autoradiography. As shown in **Figures 3A, B**, the S/preS1^16-42^ protein is recognized by both anti-S and anti-preS1 antibodies and has the highest expression level among the tested constructs, in agreement with the western blot analysis. This indicates that the aa 16-42 preS1 epitope is well displayed by the AGL, while the overall conformation of this domain is preserved in the chimeric protein. Notably, analysis of intra- and extracellular protein levels after 3 h of chase also indicates improved expression and secretion of the S/preS1^10-36^ protein when compared with the previously produced antigen, albeit at a lower level than S/preS1^16-42^.

Secretion of the S/preS1 antigens was further quantified by ELISA, which is based on recognition of the HBsAg by conformation-dependent antibodies (**Figure 3C**). The analysis indicated a significant accumulation of the S/preS1^16-42^ antigen as compared with the other chimeric proteins, thereby confirming the results of the pulse-chase experiments. Taken together, these properties qualify the S/preS1^16-42^ protein as a promising antigen candidate for further immunological studies.

### Purification of HBV-S and -S/preS^16-42^ antigens produced in mammalian cells

**Figure 4:**
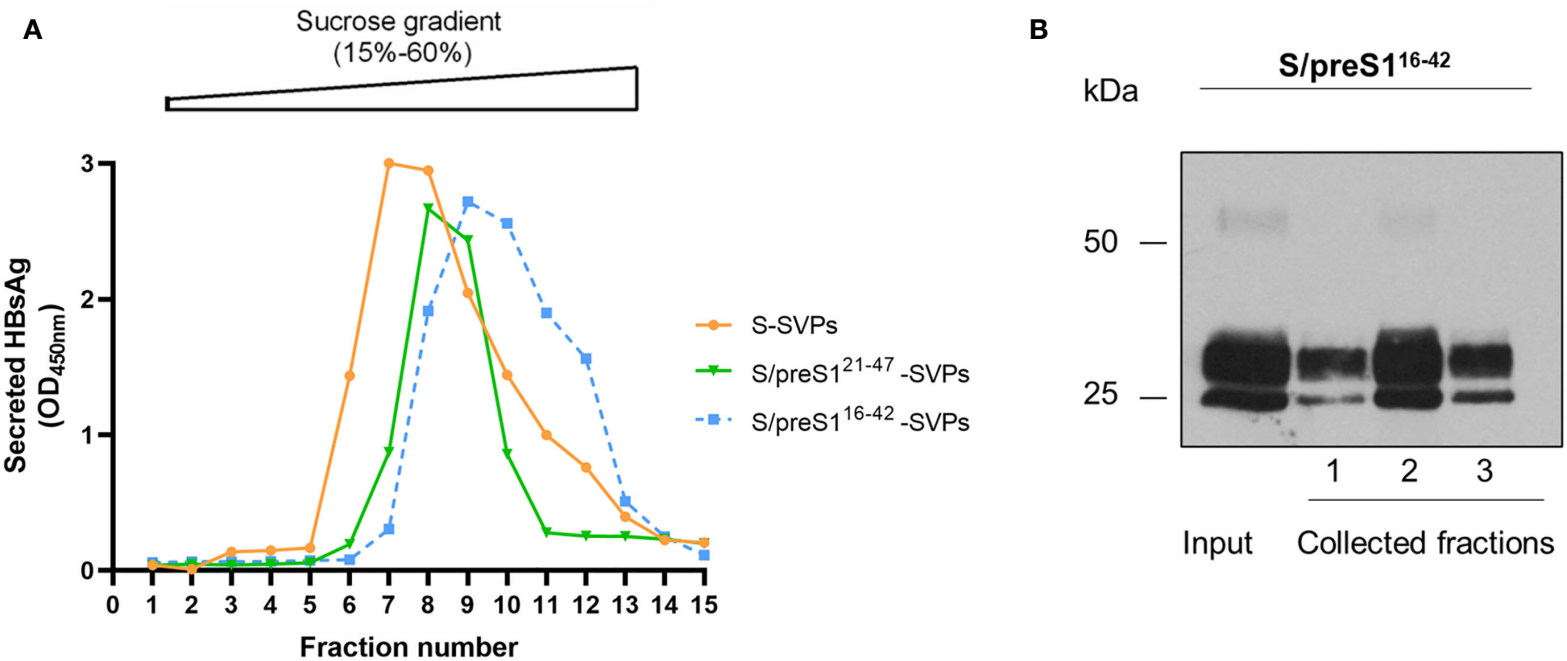
Assembly and purification of the novel S/preS1^16-42^ antigen. **(A)** HEK293T cells were transfected with pCi plasmids encoding the S/preS1^21-47^, S/preS1^16-42^, and S antigens. Cell supernatants were collected between days 4 and 10 post-transfection, 20-fold concentrated and subjected to ultracentrifugation onto a 15-60% step sucrose gradient. Collected fractions were diluted 1000-fold and analyzed for their HBsAg content by ELISA. Results are shown as optical density values measured at 450 nm. **(B)** The S/preS1^16-42^ antigen was further purified using the CaptoCore 400 resin, by gravitational flow. A representative image confirming the presence of the antigen in input sample and collected fractions, as determined by western blot using anti-preS1 antibodies is shown (n=3).

Supernatants of HEK293T cells expressing the chimeric S/preS1^16-42^, S/preS1^21-47^ antigens or the WT S, for comparison, were first concentrated on a 20% sucrose bed to collect higher molecular weight assemblies, then subjected to rate-zonal ultracentrifugation using a 15-60% sucrose gradient. Fractions were harvested, diluted in PBS to normalize for the amount of antigen (15) and analyzed for the presence of SVPs by ELISA, as above. As shown in **Figure 4A**, the sedimentation rates of chimeric and WT particles were fairly similar, while a small delay was observed for the S/preS1^16-42^–derived peak, likely reflecting assembly of this antigen into slightly larger and/or heavier SVPs. Antigen-positive fractions were pooled, dialyzed against PBS and further purified by size-exclusion chromatography, using the CaptoCore 400 resin, which excludes large molecules (>400 kDa) from entering into the matrix pores. As this resin contains both hydrophobic and positively charged ligands, smaller-size impurities are retained onto the column, while the chimeric antigens are collected in the flow-through and no elution step is required. Antigen-containing fractions were further confirmed by western blot (**Figure 4B**), pooled and concentrated by using 100 kDa cut-off Amicon columns. The amount of purified SVPs was determined in the concentrated samples by ELISA and western-blot, as described before (15). Typically, this procedure leads to antigen purification levels of about 35%. A side-by-side comparison of the immunogenic properties of the S and S/preS1^16-42^ antigens was further performed *in vivo*.

### The novel S/preS1^16-42^ antigen induces stronger humoral immune response and a different pattern of T cell activation as compared with the S protein.

**Figure 5:**
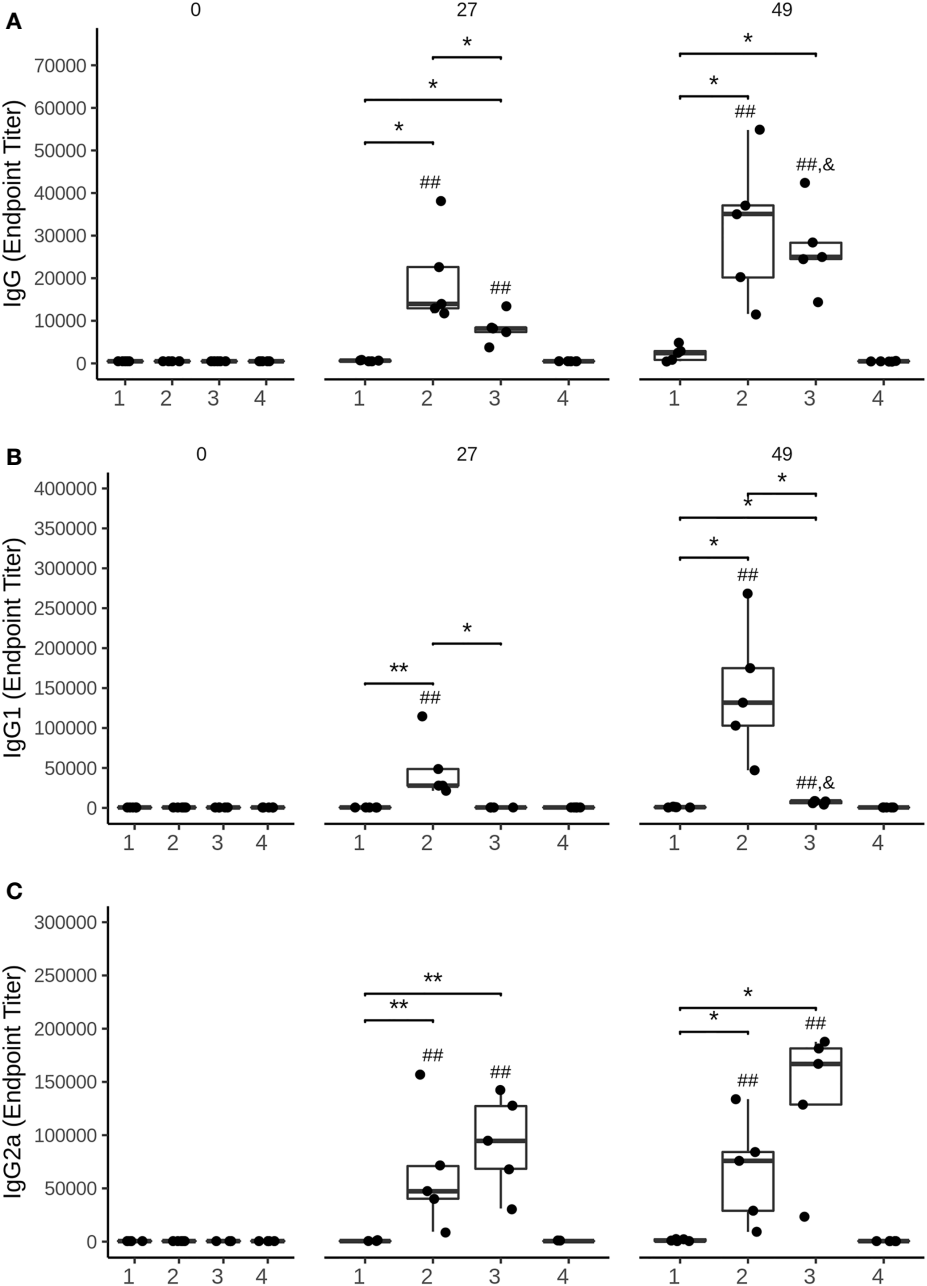
Analysis of the humoral immune response elicited by mammalian cell-derived HVB-S/preS1^16-42^ and HVB-S antigens. **(A-C)** Group of five mice were immunized 3 times, at 14-day intervals with HEK293T background protein (1), HVB-S/preS1^16-42^ (2), HBV-S (3) and Al(OH)_3_ adjuvant only (4). Antibody endpoint titres, **(A)** IgG, **(B)** IgG1 and **(C)** IgG2a, at 0, 27 and 49 days post-immunization were calculated based on a 4-parameter logistic regression curve fitted to a pool of immune sera, as the reciprocal sample dilution that would results in three times baseline + standard error as derived from the internal standard curve by multiplication (n=5). Statistical analysis was performed by using the Wilcoxon rank-sum test. Comparisons between groups at the same time point (*, p < 0.05; **, p < 0.01), time points at days 27 and 49 compared to day 0 (##, p < 0.01) and time point at day 49 compared to day 27 (&, p < 0.05) are shown.

**Figure 6:**
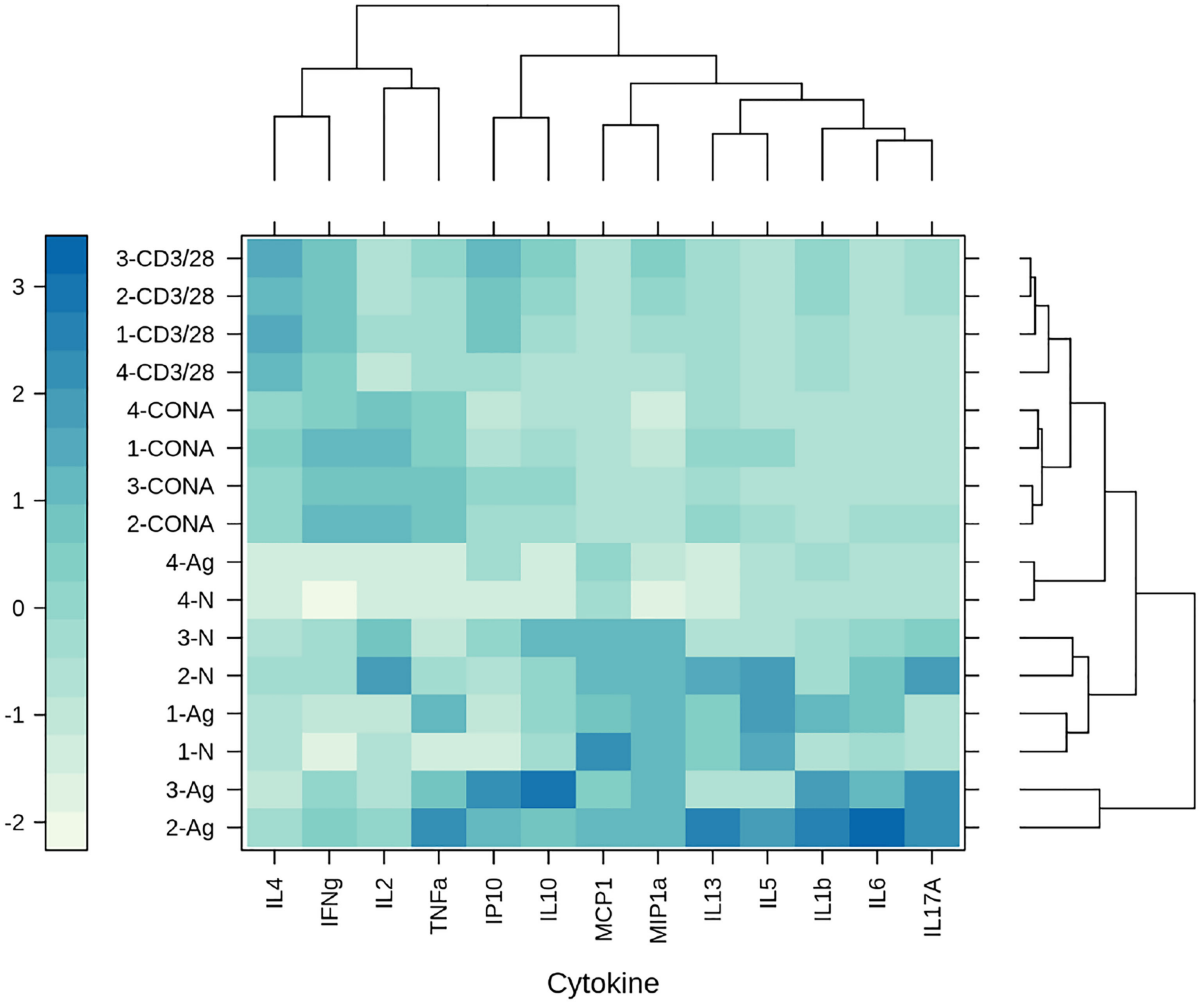
The clustered heatmap of the cytokine secretion triggered by mammalian cell-derived HVB-S/preS1^16-42^ and HVB-S antigens. Group of five mice were immunized 3 times, at 14-day intervals with HEK293T background protein (1), HVB-S/preS1^16-42^ (2), HBV-S (3) and Al(OH)_3_ adjuvant only (4). Cytokine levels were measured in spleen cells harvested from mice of each group (1-4) after stimulation with UV-inactivated HBV (Ag) and compared with unstimulated cells (N). Concanavalin A (CONA) and CD3/CD28 antibodies (CD3/28) were added as positive controls for the *in vitro* stimulation assay (n=5). Clusters and dendrograms were automatically generated, by using the hclust - manhattan and wald.D2 methods, respectively, in Rstudio (version 1.4.1103, the latticeExtra and dendextend packages). Columns and rows of the heatmap matrix show the analyzed cytokines and the stimulation agents, respectively. The blue and white colours indicate high and low levels of cytokine secretion, respectively.

The immune response triggered by the HBV-S/preS1^16-42^ and the HBV-S antigens produced HEK294T cells was investigated in Balb/c mice immunized with three doses of either protein in the presence of Al(OH)_3_, at 14-day intervals. Control lots of mice immunized with HEK293T cell supernatant-derived background proteins (group 1) or the adjuvant only (group 4) were also included in the experiment. Notably, the S/preS1^16-42^ antigen (group 2) induced an earlier and stronger IgG immune response than did S (group 3), (p < 0.05 on day 27, **Figure 5A**). Moreover, analysis of the IgG subclasses indicated significantly higher IgG1 titers in mice vaccinated with the S/preS1^16-42^ antigen (**Figure 5B**). This is consistent with the IgG1-dominant immune response induced by the former chimeric antigen, S/preS1^21-47^, suggesting activation of Th2 cells and consequently, of the humoral immunity (15). Both S and S/preS1^16-42^ were also able to elicit significant titers of IgG2a antibodies, an indication of potential Th1 cell-mediated activation of cellular immunity (**Figure 5C**). Interestingly, the yeast-derived S protein was shown to induce low levels of this IgG subclass (33) and a similar result was obtained in our previous study following immunization with mammalian cell- or plant-derived HBV-S protein in the absence of adjuvants (15). To analyze in more detail a possible activation of the cellular immune response by adjuvanted-HBV antigens produced in HEK293T cells, a cytokine-multiplex immunoassay was performed to quantify secretion of relevant cytokines from splenocytes of vaccinated mice. The clustered heatmap resulting from this analysis indicates a broad spectrum of HBV-reactive T cells generated by immunization with the S and S/preS1^16-42^ antigens (**Figure 6**). The complexity of this immune response is illustrated by the presence of pro- (TNF-α, IL-1β, IL-6, IL-17A) and anti-inflammatory cytokines (IL-10) (34), some of them with known antiviral activity against HBV (IL-1β, IL-6, IL-17A) (35–37). Importantly, both immunogens induced secretion of IFN-γ, the well-accepted marker of Th cell activation, above the levels found in controls; however, this was not the predominant cytokine (**Figure 6**). This observation is in line with the previously published data showing that IFN-γ is underrepresented amongst the cytokines elicited in humans vaccinated with the commercial HBV vaccine (38). Although the quantification of more specific mediators of cellular immunity, such as IL-4 and IL-2 was obscured by the higher background in non-stimulated samples, overall, the results indicate evident T cell activation, consistent with the presence of IgG2a antibodies in mice immunized with either the S or the S/preS1^16-42^ antigen. Notably, there is little overlap between the cytokine patterns induced by either HBV antigens, suggesting stimulation of different T cell subsets.

### Immunization with the S/preS1^16-42^ antigen triggers both anti-S and anti-preS1 antibodies

**Figure 7:**
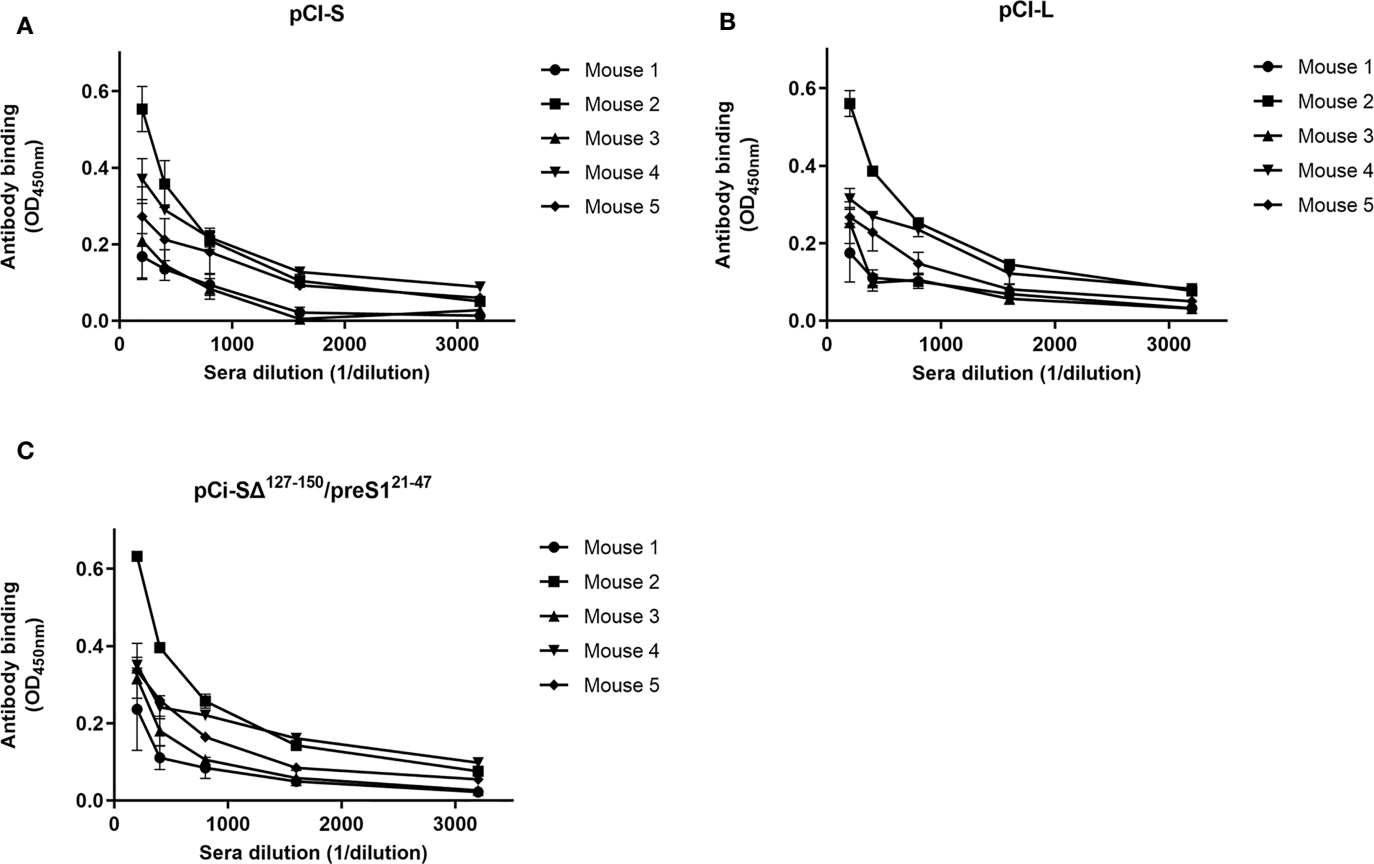
Binding of antibodies elicited by the HVB-S/preS1^16-42^ antigen to HBV surface proteins. Lysates of HEK293T cells transfected with pCi plasmids encoding for the S, **(A)**, L **(B)**, and SΔ^127-150^/preS^121-47^ **(C)** proteins were used to coat 96-well plates. Serial dilutions of pooled pre-immune sera and of sera from mice immunized with S/preS1^16-42^ were added to the plates, followed by incubation with HRP-conjugated anti-mouse secondary antibodies. Antibody binding is shown as optical density values measured at 450 nm following subtraction of those obtained for the pre-immune sera (n=3). Data are represented as means ± SD for each individual mouse.

To determine the specificity of antibodies induced by the S/preS1^16-42^ antigen, the binding of sera from immunized mice to the S and L proteins was tested by ELISA. As the L protein also contains the S domain at the C-terminus, antibody reactivity with the SΔ^127-150^/preS1^21-47^ protein was tested. This chimeric protein lacks the AGL domain and hence reactivity to anti-S antibodies, while it strongly binds preS1 antibodies, due to the display of the aa 21-47 sequence, as previously documented (15). As shown in **Figure 7**, the sera from all mice immunized with the S/preS1^16-42^ antigen reacted similarly with L, S and SΔ^127-150^/preS1^21-47^. This indicates that both S and preS1 epitopes are equally well presented to the immune system cells, highlighting the capacity of this novel chimeric antigen to trigger a mixture of anti-S and -preS1 antibodies.

### Immunization with the S/preS1^16-42^ antigen induces antibodies with neutralizing activities against both WT and a vaccine escape mutation HBV variant

**Figure 8:**
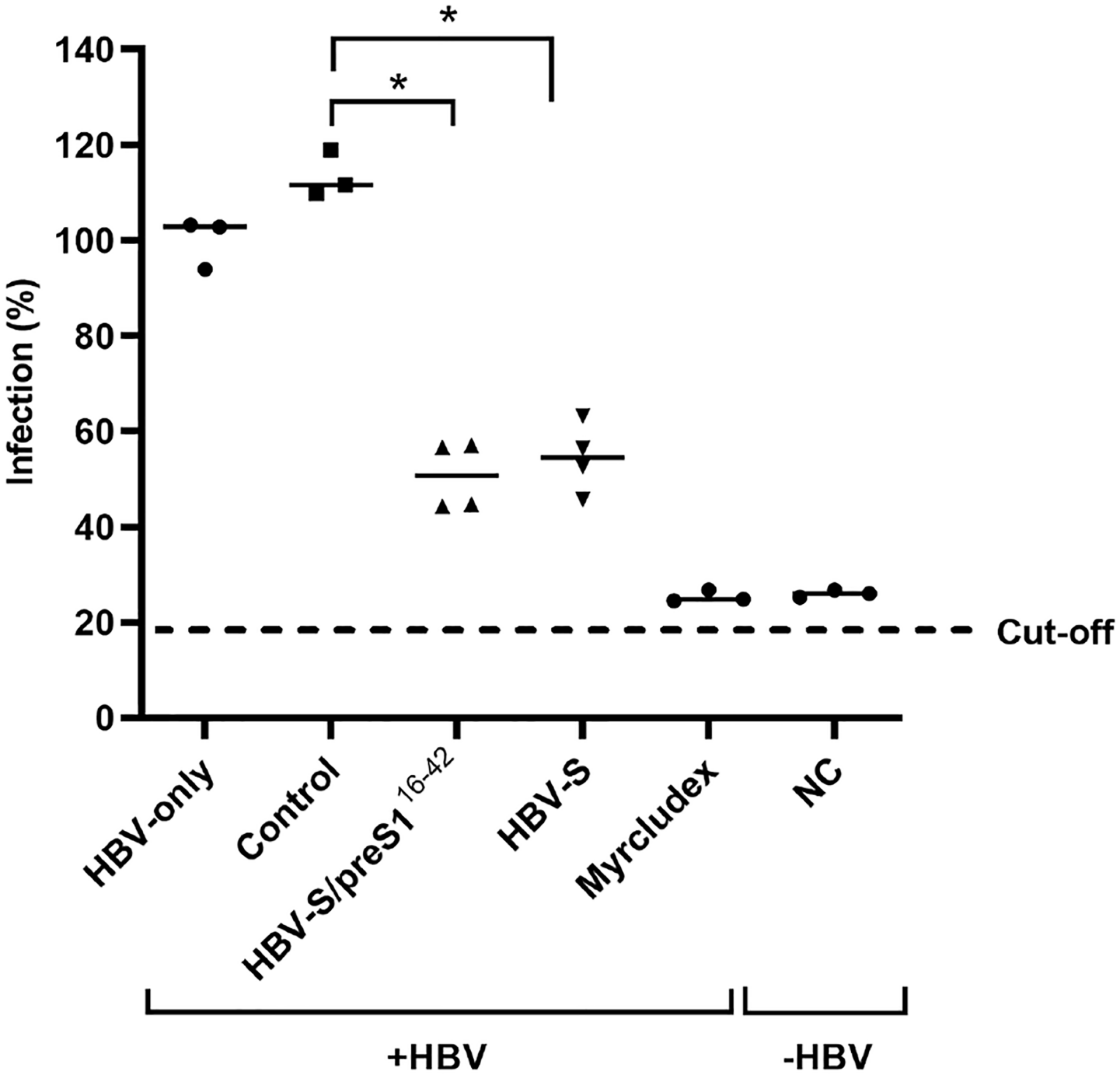
Neutralization of WT HBV infection by S and S/preS1^16-42^ antisera. Pooled pre-immune sera or sera from mice immunized with S, S/preS1^16-42^, or background proteins (control) were diluted 1:50 and pre-incubated with HBV inoculum (100 Geq/cell), in medium supplemented with 4% PEG, for 1 h. HepG2^hNTCP^ cells were incubated with the sera-treated HBV inoculum for 16 h, or maintained non-infected, as negative control (NC). Cells incubated with Myrcludex B for 3 h prior to infection were used as a control for the specificity of inhibition of HBV infection. At 16 h post-infection the medium was supplemented with 2.5% DMSO and then changed every two days. Cell media were collected at day 11 post-infection and used to quantify the HBeAg levels by ELISA. Data are shown as percentage of HBV infection in the presence of post-immune sera from infection values obtained in the presence of the pre-immune sera, at the same dilution (n=4). Values in the presence of Myrcludex B and in the negative-control represent percentages of infection from HBV-only samples (n=4). Statistical analysis was performed by using the Mann-Whitney U test. Comparisons between control and HBV-S/preS1^16-42^ or S groups are shown (*, p < 0.05).

To comparatively determine the ability of the immune response induced by the S and the S/preS1^16-42^ antigens to protect against infection, HBV particles were incubated with pre- or post-immunization sera (1:50 dilution) from mice vaccinated with either antigen or HEK293T cell background proteins (control), before infection of permissive HepG2^hNTCP^ cells (100 GEq/cell). Cells treated with Myrcludex B prior to infection were also used as an additional control for specific inhibition of HBV infection. Quantification of the HBeAg secreted in cell media was performed by ELISA and resulting values were normalized against those obtained for the pooled pre-immune sera of each group. Notably, the sera from mice immunized with either HBV antigen significantly inhibited HBV infection, when compared with controls (**Figure 8**).

**Figure 9:**
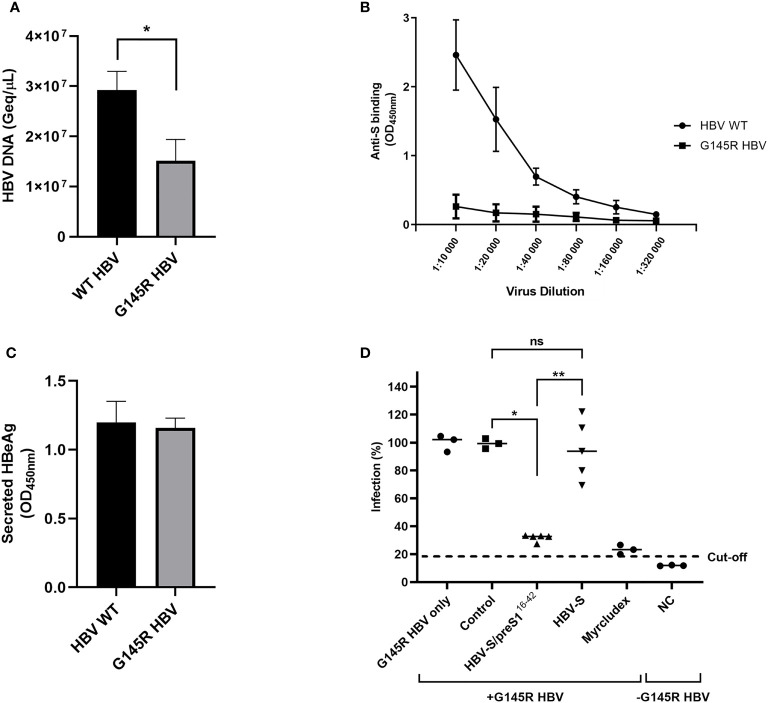
Neutralization of G145R HBV infection by S and S/preS1^16-42^ antisera. **(A)** Huh7 cells were transfected with pGEM-4Z-HBV 1.3 WT or pGEM-4Z-HBV 1.3 G145R. Secretion of HBV particles was quantified in cell media by qPCR at day 7 post-transfection (n=4). Data are represented as means ± SD; Student’s unpaired t test (*, p < 0.05). **(B)** Serial dilutions of WT and G145R HBV particles were incubated with anti-S antibodies and specific binding was quantified by using the Monolisa HBsAg Ultra kit. Results are shown as optical density values measured at 450 nm (n=3). Data are represented as means ± SD. **(C)** HepG2^hNTCP^ cells were incubated with WT and G145R HBV particles (100 Geq/cell) for 16 h in the presence of 4% PEG. At 16 h post-infection the medium was supplemented with 2.5% DMSO, then changed every two days. Cell media were collected at day 11 post-infection and used to quantify the HBeAg levels by ELISA. Results are shown as optical density values measured at 450 nm (n=3). Data are represented as means ± SD. **(D)** Pooled pre-immune sera or sera from mice immunized with S, S/preS1^16-42^, or background proteins (control) were diluted 1:50 and pre-incubated with G145R HBV inoculum (100 Geq/cell), in medium supplemented with 4% PEG, for 1 h. HepG2^hNTCP^ cells were incubated with the sera-treated HBV inoculum for 16 h, or maintained non-infected, as negative control (NC). Cells incubated with Myrcludex B for 3 h prior to infection were used as a control for the specificity of inhibition of HBV infection. At 16 h post-infection the medium was supplemented with 2.5% DMSO and then changed every two days. Cell media were collected at day 11 post-infection and used to quantify the HBeAg levels by ELISA. Data are shown as percentage of HBV infection in the presence of post-immune sera from values obtained in the presence of the pre-immune sera, at the same dilution (n=5). Values in the presence of Myrcludex and in the negative-control represent percentages of infection from G145R HBV-only samples (n=5). Statistical analysis was performed by using the Mann-Whitney U test. Comparisons between control and either antigen groups and between the antigen groups are shown (*, p < 0.05; **, p < 0.01; ns, no statistical significance).

The AGL domain is composed of two loops stabilized by intra-molecular disulfide bonds and the second loop, spanning the 139 to 149 aa sequence of the S protein is the major target of HBV-neutralizing antibodies induced by current S-based vaccines (39). Antibody binding is extremely sensitive to conformational changes of this region; therefore, mutations affecting the AGL folding result in selection of vaccine escape HBV variants (40). To determine the ability of S and S/preS1^16-42^ antisera to neutralize a vaccine escape mutant, the most relevant G145R mutation (41–43) was introduced in the HBV sequence and HBV-S^G145R^ viral particles were produced in Huh7 cells. PCR-quantification of the virus load revealed impaired secretion of HBV-S^G145R^ particles, in agreement with previous reports (44, 45) (**Figure 9A**). Equal amounts of the WT and the mutant HBV were serially diluted before analysis by HBsAg ELISA. As expected, the WT HBV particles were efficiently recognized by the highly conformation-dependent anti-S antibodies, in contrast to the HBV-S^G145R^ variant, confirming the deleterious effect of the G145R mutation on the AGL structure (**Figure 9B**). The potential consequence of the G145R mutation on the HBV infectivity was further assessed following infection of HepG2^hNTCP^ cells with equal amounts of WT and mutant viral particles and quantification of the secreted HBeAg. As shown in **Figure 9C**, infection occurred with similar efficiency regardless of the presence of the vaccine escape mutation. The neutralization of HBV-S^G145R^ infection by the S and S/preS1^16-42^ antisera was next investigated using the same experimental procedure and controls as for the WT virus. Notably, the sera from mice immunized with the S/preS1^16-42^ antigen significantly inhibited HBV-S^G145R^ infection, at levels similar to those achieved by the Myrcludex treatment (**Figure 9D**). A more heterogeneous response was observed in the group of mice immunized with the S antigen and no significant neutralization capacity when compared with the control group (**Figure 9D**). This is consistent with the notion that antibodies elicited by the WT S immunogen do not efficiently prevent infection with HBV bearing the G145R mutation.

## Discussion

The HBV vaccine has made considerable contributions to the control and management of HBV infection. However, the shortcomings encountered in the past decades have shown that novel antigens with improved immunogenicity are needed. Owing to its crucial role in HBV infection, the preS1 domain of the L envelope protein has been the focus of many studies aiming to solve the issues associated with the S only-based vaccines (46). Indeed, recent data have shown that antibodies targeting the preS1 domain have robust neutralizing activity against HBV and protect humanized mice from infection (32). Moreover, preS1 antibodies are able to clear the viral particles in HBV-carrier mice, a strong indication that the preS1 region is also a T cell activator and thus could play a major role in breaking the immune tolerance to HBV (47). With the emerging studies revealing the exciting properties of the preS1-targeting antibodies, various approaches have been considered to produce the best vaccine formulation able to trigger such an immune response. This intense research has led to the development of third-generation vaccines that are based on the production of SVPs containing all three surface proteins, S, M and L, in mammalian cells (12, 48). These vaccines had favorable safety profiles in clinical trials and achieved earlier and higher seroprotection rates when compared with second-generation, yeast-derived vaccines, even in the specific risk-groups of non-responders (12, 49).

A promising alternative to using the whole L protein as an immunogen is to expose only the relevant, virus neutralizing preS1 epitopes on the surface of the S protein. Such chimeric proteins would have the advantage of displaying equimolar ratios of preS1 and S epitopes on the S protein backbone, as opposed to the poor incorporation rate of the L protein in mixed SVPs (50–52). The ability of S-SVPs to carry heterologous sequences has previously been demonstrated by the successful development of chimeric antigens containing clinically relevant epitopes derived from the human malaria parasite *P. falciparum* or other viruses, such as HCV and polio (53–56). These recombinant proteins triggered high titers of neutralizing antibodies against the foreign epitopes and also retained the ability to mount a humoral immune response specific for the carrier S protein, albeit at lower levels when compared with the standard vaccine (55, 57). Our own studies demonstrated the superior immunogenic properties of the S/preS1^21-47^ chimera produced in mammalian cells and plants, when compared with the S protein. This antigen was able to induce systemic and mucosal immune responses in mice immunized by parenteral and oral administration, respectively (15, 20).

In this report we aimed to refine the design and production of S/preS1 chimeric antigens by inserting novel epitopes spanning the preS1 domain, within the AGL domain of the S protein and exploring the quality of the humoral and cellular immune response elicited by the resulting recombinant proteins. Of the four antigen candidates generated in this study, S/preS1^16-42^ had significantly higher expression and secretion levels as compared with the previously developed S/preS1^21-47^ chimera and exhibited reactivity to both anti-preS1 and anti-S antibodies. Therefore, this protein was selected for a detailed immunological analysis *in vivo*. An earlier onset of antibody production was observed in mice immunized with S/preS1^16-42^, which appeared after the second injection, as compared with those receiving the S protein, accompanied by significantly higher IgG titers. Interestingly, a recent HBV vaccine clinical trial performing a head-to-head assessment of the efficacy of the 3-antigen formulation containing the L, M and S proteins produced in mammalian cells and the standard, S only-based vaccine, indicated a higher rate of seroprotection achieved by the former (49). These results confirm the hypothesis that incorporation of preS sequences in novel HBV vaccines is likely to increase their immunogenicity and efficacy.

The S/preS1^16-42^ immunogen induced a mixture of anti-preS1 and anti-S antibodies, indicating efficient presentation of the novel preS1 epitope by the AGL domain. It is likely that the insertion of the preS1 sequence between aa126 and 127 of the S protein did not significantly perturb the stability of the AGL domain due to extensive intramolecular disulfide bonding within this region. These antibodies belong to both IgG1 and IgG2a subclasses, unlike those elicited by the S protein that are enriched in IgG2a, suggestive of different T-cell activation and signaling triggered by the two antigens. Although traditionally it is inferred that a Th2-type immune response is preferentially primed by Al(OH)3- adjuvanted antigens, the intramuscular administration route can activate both Th1 and Th2-mediated pathways, also depending on the antigen structure (58). Quantitative analysis of the cellular immune response, confirmed this observation, revealing predominant induction of TNF-α, IL-13 and IL-6 in mice immunized with the S/preS1^16-42^ antigen as opposed to IL-10 and IP-10, the main cytokines induced by S. Due to their adjuvantation with aluminum hydroxide, a known activator of the inflammasome pathway (59, 60), both antigens induced IL-1β, at similar levels.

Although the cell-mediated immunity to HBV vaccination is less well understood, this response is considered important for efficient control of HBV infection (61). It has been shown that TNF-α, a strong pro-inflammatory cytokine, activates proliferation of HBV-specific cytotoxic T lymphocytes (62) and counteracts suppression of this response by regulatory T cells (63). Surprisingly, TNF-α rather than IFN-γ is the predominant cytokine secreted in humans after vaccination with the S-based HBV vaccine produced in yeast (38). Arguably, an increase in TNF-α-producing T cells may indicate a better immunization outcome in both healthy individuals and risk groups, as recently shown (64, 65). Moreover, TNF-α inhibits HBV DNA replication at nontoxic concentrations for the host cell, by a mechanism requiring NF-κB-signaling, which may contribute to an early suppression of HBV infection, should this occur in patients with low anti-HBV antibody titers (66). Similarly, IL-6 inhibits HBV transcription by epigenetic mechanisms, in addition to performing a broad range of pro- and anti-inflammatory signaling (36). This pro-inflammatory cytokine pattern is similar to that resulted after the viral infection or TLR-9 stimulation, suggesting a crosstalk between that innate and adaptive immunity influencing the antigen specific immune response (34, 67). Clearly, further research involving more relevant animal models is needed to understand the true benefit of a particular T-cell activation pattern. This will ultimately depend on the fine balance between the capacity of the complex cytokine-mediated pathways to boost anti-HBV immunity on one hand, and to potentially cause liver inflammation, on the other (34).

An important finding emerging from this study is the equivalence of the S and S/preS1^16-42^ antigens produced in mammalian cells in terms of activating an efficient WT HBV-neutralizing humoral immune response and the evident superiority of the latter immunogen to prevent infection with the G145R vaccine-escape mutant. This is the most stable and frequent mutation identified in vaccinated children (42, 68) and adults under immunosuppressive therapy (69, 70). The recent use of high-resolution DNA sequencing technologies suggests that in fact, the frequency of HBV mutations may have been largely underestimated. Notably, mathematical models of epidemiological data indicate that the G145R mutation hampers HBV diagnosis and has spreading potential, supporting timely modifications of current vaccines in order to protect against this HBV variant (71). The ability of anti-preS1 antibodies to protect against the G145R vaccine escape mutant has recently been documented in a reporter-based HBV infection assay (24). While our results using cell culture HBV particles are in line with this report, the ability of the S/preS1^16-42^ antisera to neutralize the G145R variant more efficiently than the WT HBV is intriguing, since both particle types have the same preS1 sequence and are equally infectious. Although this difference may simply reflect an experimental variation, it is tempting to hypothesize that the S/preS1^16-42^ antigen may trigger a different set of anti-S antibodies that retain the ability to bind the G145R mutated AGL, thus contributing to this strong neutralization effect. Future work will attempt to sequentially deplete the S/preS1^16-42^ antiserum of either anti-S or anti-preS1 antibodies, followed by analysis of their capacity to bind and neutralize WT and G145R HBV variants, to clarify the quality of the immune response induced by chimeric S/preS1 antigens. Nevertheless, our results indicate that combining relevant epitopes in one molecule is a valid strategy to increase activation of the immune response and expand its specificity and thus improve efficiency of HBV vaccination.

## Data availability statement

The original contributions presented in the study are included in the article/supplementary material. Further inquiries can be directed to the corresponding authors.

## Ethics statement

The animal study was reviewed and approved by ANSVSA, Romania.

## Author contributions

Conceived and designed the experiments: NB-N, A-MP, M-OD, CL, JL-C, CS. Performed the experiments: A-MP, M-OD, CSc, IC, II, CT, AC, CSt. Analyzed the data: NB-N, A-MP, CSc, AO, CT, IC, II, AC. Wrote the paper: NB-N, A-MP, JL-C, CSt. All authors approved the submitted version of the manuscript.

## Funding

This research has received funding from the EEA Grants 2014-2021, SmartVac project no. 1SEE/2019. A-MP was supported by a PhD fellowship of the Romanian Academy. The funding institution had no role in study design, the collection, analysis and interpretation of data, the writing of the report or the decision to submit the article for publication.

## Conflict of interest

The authors declare that the research was conducted in the absence of any commercial or financial relationships that could be construed as a potential conflict of interest.

## Publisher’s note

All claims expressed in this article are solely those of the authors and do not necessarily represent those of their affiliated organizations, or those of the publisher, the editors and the reviewers. Any product that may be evaluated in this article, or claim that may be made by its manufacturer, is not guaranteed or endorsed by the publisher.
